# Vitamin D and brain volumetric changes: An updated systematic review and meta-analysis

**DOI:** 10.1016/j.ibneur.2025.04.011

**Published:** 2025-04-27

**Authors:** Rozhina Tamannaeifar, Salar Yousefzadeh, Sana Rahmani, Maedeh Bayani, Mahdiyeh Nozad Varjovi, Nima Eftekhari, Mona Ranjkesh, Mahsa Kohansal Vajargah, Sajjad Hajihosseini, Faezeh Ahanj, Hadis Sarlak, Komeil Aghazadeh-Habashi, Melika Arab Bafrani, Alaleh Alizadeh, Yaser Khakpour, Niloofar Deravi

**Affiliations:** a Bioprocessing and Biodetection Laboratory, Department of Food Science and Technology, University of Tehran, Tehran, Iran; b Student Research Committee, Qazvin University of Medical Sciences, Qazvin, Iran; c Faculty of Medicine, Iran University of Medical Sciences, Tehran, Iran; d Student Research Committee Shahid Beheshti University of Medical Sciences, Tehran, Iran; e Student Research Committee, Faculty of Pharmacy, Tabriz University of Medical Sciences, Tabriz, Iran; f Qazvin University of Medical Science, Qazvin, Iran; g Babol University of Medical Science, iran; h Nephrology Section, Department of Medicine, Hasheminejad Kidney Center, School of Medicine, Iran University of Medical Sciences, Tehran, Iran; i Student Research Committee, Tehran University of Medical Sciences, Tehran, Iran; j Faculty of Iranian Medicine, Tabriz University of Medical Sciences, Tabriz, Iran; k Iran University of Medical Sciences (IUMS), School of Public Health, Iran; l Student Research Committee, Tabriz University of Medical Sciences, Tabriz, Iran; m Students' Scientific Research Center (SSRC), Tehran University of Medical Sciences, Tehran, Iran; n Student Research Committee, Faculty of Medicine, Mashhad Branch, Islamic Azad University, Mashhad, Iran; o Faculty of Medicine, Guilan University of Medical Sciences, Rasht, Iran; p Student Research Committee, School of Medicine, Shahid Beheshti University of Medical Sciences, tehran, Iran

**Keywords:** Vitamin D, Brain Volumetric Changes, Gray Matter, Meta-Analysis

## Abstract

**Background:**

In this systematic review and meta-analysis, we aimed to investigate the relationship between vitamin D levels and brain volumetric changes in human studies.

**Method:**

We conducted a comprehensive search in PubMed, Scopus, and Google Scholar databases up to December 2024. A total of 450 studies were identified. Following title, abstract, and full-text screening, we included three studies for analysis. Data were extracted from these studies and analyzed using appropriate statistical methods.

**Result:**

Our analysis revealed that the included case-control and cohort studies were conducted in the United States, Norway, and the Netherlands. The studies exhibited a range of characteristics, including sample size (number of patients: 183–240), demographic variables, and methods of assessing both vitamin D levels and brain volume. Brain volume assessments included gray matter, white matter, and total brain volume. The total follow-up duration across studies was 11 years. The age of participants ranged from 30 to 64 years in one study, while in another, they were aged 65 years or older. The meta-analysis indicated no significant association between vitamin D levels and brain volumetric changes across the included studies (effect size: 0.07, 95 % CI= [-0.01, 0.15], P = 0.07, I^2^=54.44 %).

**Conclusion:**

This meta-analysis did not establish a significant association between vitamin D levels and brain volumetric changes. These findings highlighted the need for further large-scale studies to clarify the potential role of vitamin D in brain volume and to better understand the underlying mechanisms involved.

## Introduction

Vitamin D is conventionally recognized as a crucial agent for bone metabolism; however, it has recently gained attention for its pleiotropic biological effects in neurobiology. Its actions are mainly mediated through Vitamin D Receptors (VDRs), which are ubiquitously expressed in various tissues, including neurons (Derks et al., 2008). The presence of VDRs in the brain suggests that vitamin D might modulate processes beyond metabolism, potentially related to the maintenance of cognitive health (Anjum et al., 2018). The VDRs are encoded by the VDR gene, which is located on human chromosome 12q13.11 (Bastyte et al., 2023). The VDR gene is expressed in both neurons and glial cells, especially in the cortex and hippocampus (Eyles et al., 2005). VDR upregulates neuroprotective genes such as BDNF and SOD2, and downregulates apoptotic factors like BAX (Quialheiro et al., 2023, Zahra et al., 2024). Common VDR polymorphisms modulate these pathways; however, further study is required (Retraction, 2025). Vitamin D exhibits neuroprotective properties, helping to regulate neurotrophic factors, antioxidants, and anti-inflammatory agents—key elements crucial for neuronal health and cognitive functioning (Harms et al., 2011, Moore et al., 2005).

Emerging evidence from recent studies has shown a significant relationship between serum levels of 25-hydroxyvitamin D (25OHD) and brain volumetric measures in older adults (Annweiler et al., 2014). Lower serum 25OHD concentrations have been associated with morphological brain changes thought to predispose individuals to cognitive decline and to increase the risk of neurodegenerative diseases, including Alzheimer's disease (Feart et al., 2017). Despite these findings, limited information exists about the specific brain regions affected by vitamin D deficiency.

Research into the neurobiological roles of vitamin D is significant, given that approximately 1 billion people worldwide are estimated to have insufficient levels of 25OHD, with older adults affected most prominently. In this demographic, the prevalence of vitamin D insufficiency ranges between 40 % and 90 %. Notably, hypovitaminosis D has been associated with several adverse health consequences, such as cognitive impairment and an increased risk of dementia (Byrn and Sheean, 2019). Indeed, studies have reported that low circulating levels of vitamin D are related to poorer cognitive performance, particularly in executive function and episodic memory (Miller et al., 2015). Contrarily, higher serum vitamin D levels were associated with improved cognitive outcomes and a reduced risk of neurodegenerative disorders (Annweiler et al., 2015).

The relationship between vitamin D and neuroanatomy becomes even more interesting when examined through the lens of imaging studies. Some investigations have suggested an inverse relationship between vitamin D and intracranial volume; however, most studies focused on older populations (Annweiler et al., 2014). Therefore, the relation between volumetric changes and vitamin D levels deserve further exploration, especially about their cognitive implications. Given that VDRs are expressed in key brain regions, such as the hippocampus and cortex – areas critical for learning and memory, the effects of vitamin D on brain structure and function are of significant interest (Eyles et al., 2005).

The purpose of this systematic review was to delve into the available literature regarding the relationship between vitamin D status, brain volumetric changes, and cognitive decline. This review aims to provide updated insights into the neuroanatomical implications of vitamin D deficiency and its potential to act as a modifiable risk factor for cognitive decline.

## Material and method

This research involved a systematic review and meta-analysis to investigate the relationship between vitamin D (25-hydroxyvitamin D) and brain volumetric changes. The methodology was conducted in accordance with the PRISMA (Preferred Reporting Items for Systematic Reviews and Meta-analyses) guidelines (Moher et al., 2009). Additionally, the research protocol for this study was registered with PROSPERO.

## Literature search

An advanced search strategy was employed to identify relevant articles from the electronic databases of Google Scholar, PubMed/Medline, and Scopus. The search focused on the primary Medical Subject Headings (MeSH) associated with Vitamin D (25-Hydroxyvitamin D 2) and Brain Volumetric Changes. Search terms were applied to the title or abstract, and the strategy was tailored to the specific query formats of each database (Table 1). No limitations were imposed on publication date, type, or language. To ensure not missing relevant articles, reference lists from previous systematic reviews and included studies were also screened. This approach effectively reduced the risk of overlooking significant articles while facilitating the retrieval of relevant studies. The screening process was conducted independently by two reviewers, with discrepancies resolved through discussions among the authors.

**Table 1:** Search strategy through various databases.

Search Engine	Search Strategy	Date & Number
PubMed	((Vitamin D[Title/Abstract]) OR (25-Hydroxyvitamin D 2[Title/Abstract])) AND ((brain volumetric[Title/Abstract]) OR (brain volume[Title/Abstract]))	8/4/2024 27 results
Scopus	(TITLE-ABS-KEY (vitamin d) OR TITLE-ABS-KEY (25 hydroxyvitamin d2) AND TITLE-ABS-KEY (brain volumetric) OR TITLE-ABS-KEY (brain volume))	8/4/2024 394 results
Google Scholar	allintitle: Vitamin D brain volumetric	8/4/2024 2 results
	allintitle: Vitamin D brain volume	8/4/2024 9 results
	allintitle: 25-Hydroxyvitamin D 2 brain volumetric	8/4/2024 0 results
	allintitle: 25-Hydroxyvitamin D 2 brain volume	8/4/2024 0 results

## Criteria for selecting studies

To qualify for inclusion in this meta-analysis, studies were required to meet the following criteria:

1) The methodology should focus on observational studies while effectively controlling for confounding variables related to any interventions.
2) The primary aim of the study must be to evaluate the correlation between vitamin D (25-hydroxyvitamin D) and brain volumetric changes.
3) The participant group should consist exclusively of individuals diagnosed with brain volumetric change.
4) Definitions of brain volumetric changes must align with the specific study design employed.

Studies using alternative methodological approaches, such as experimental in vivo or in vitro research, animal studies, and reports of unrelated outcomes, were excluded.

## Data extraction and study quality assessment

In order to assess the eligibility of the studies, two reviewers independently screened the titles and abstracts of identified studies based on the inclusion criteria. Studies that did not meet these criteria were excluded. Then, the full texts of the remaining studies were reviewed, and those deemed eligible underwent data extraction. Data extraction was performed across four data categories:

1) Study characteristics, including authors, study design, geographical location, and publication year.
2) Demographic variables, including the nationality and age group of the patients.
3) Study design features, including the total number of participants, study duration, and the criteria used to define brain volumetric changes.
4) Outcomes, specifically the correlation between brain volumetric changes and vitamin D (25-hydroxyvitamin D).

Two reviewers independently evaluated the quality of the included studies (cohort, case-control, and analytical cross-sectional studies) using the JBI Critical Appraisal Tool (https://jbi.global/critical-appraisal-tools). In cases of disagreement, a third reviewer was involved to facilitate resolution.

### Statistical analysis

Data analysis was conducted utilizing STATA 13.1 software. The results were presented as pooled odds ratios (ORs) with 95 % confidence intervals (CIs) and illustrated in forest plots. To assess the heterogeneity among the included studies, we employed the I^2^ statistic. A random-effects model was applied when significant heterogeneity was present (I^2^ > 50 %). Additionally, a sensitivity analysis was performed by systematically excluding one study at a time to verify the robustness of the results. Moreover, we examined potential publication bias through visual inspection of the funnel plot symmetry and Egger's regression analysis.

## Result

### Study selection

**Fig. 1:**
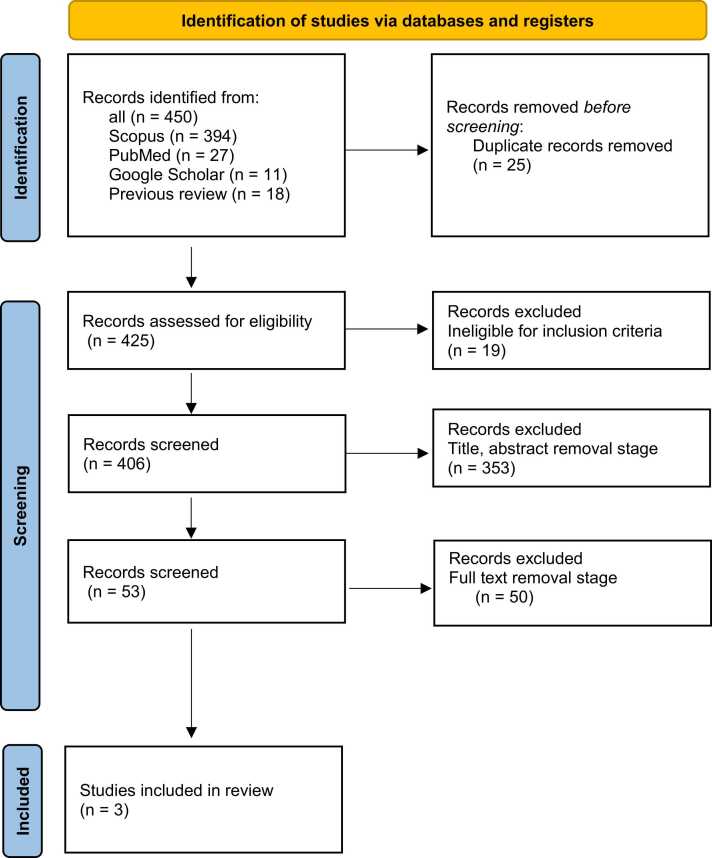
PRISMA diagram of selection process and including eligible studies.

We performed this research as an updated review of a previous article published in 2014 (Annweiler et al., 2014). A total of 450 references were identified by searching the PubMed, Scopus, and Google Scholar databases, in addition to the references of the previous review. After removing 25 duplicates, 425 studies were retrieved and underwent further evaluation. Following title/abstract and full-text screening, 413 and 9 articles were excluded, respectively. Additionally, a detailed Excel file documenting the screening and exclusion process for each study has been provided in the supplementary materials. Ultimately, three studies were included in this meta-analysis. The selection process is summarized in Fig. 1.

### Baseline characteristics

Study characteristics and quality assessment results are summarized in Table 2. We appraised three studies with two study designs: two cross-sectional studies conducted in the Netherlands (Brouwer-Brolsma et al., 2015) and Norway (Berg et al., 2018), in addition to a prospective cohort study from the USA (Beydoun et al., 2020), published in 2015, 2018, and 2020, respectively. These studies evaluated the correlation between serum 25(OH)D levels and total brain volume, gray matter volume, and white matter volume.

**Table 2:** LC-MS/MS: Liquid Chromatography-Tandem Mass Spectrometry, SPM: Statistical Parametric Mapping, VBM: Voxel-based morphometry.

						Vitamin D		Brain		
Author [ref]	Study design	Country	Patients number (% female)	Age	Inclusion criteria	Method	Measures	Method	Measures	Quality assessment
Brouwer-Brolsma et al. (Brouwer-Brolsma et al., 2015)	Cross-sectional study	Wageningen, Netherlands	217 (43 %)	72 ± 6 y	females Dutch community-dwelling; age≥ 65 y, have mild hyperhomocysteinemia	LC-MS/MS	25OHD serum concentration (Threshold: 50 nmol/L	3-Tesla MRI scanner (Siemens Magnetom Verio, Ede, Netherlands) Analyzed by VBM, using SPM8 software	Total brain volume, gray matter volume, white matter volume	8/8
Berg et al. (Berg et al., 2018)	Cross-sectional study	Oslo,Norway	184 (44 %) (psychosis: 83 / control: 101)	30.53 ± 9.42 y	European ancestry IQ*>* 70, no signs of organic etiology, speak a Scandinavian language, no severe trauma in CNS	From September 2012, the LC-MS/MS **Prior to September 2012**, the radioimmunoassay (RIA [kit from Diasorin])	Serum 25OHD concentration, used as a continuous variable; a **regression equation** (LC-MS/MS = 1.16 × RIA − 9) was developedto convert the LC-MS/MS results into equivalent RIA measurment	3-Tesla MRI scanner (General Electric Signa HDxt, Ullevål, Oslo University Hospital), Analyzed by FSPGR	Whole brain volume (mm³), White matter volume (mm³), Peripheral grey matter volume (mm³), Grey matter volume (mm³), Total ventricular volume (mm³)	8/8
Beydoun et al. (Beydoun et al., 2020)	prospective cohort study	Baltimore, USA	240 (59 %)	47.7 ± 8.9 y	age= 30–64 Not pregnant, not having cancer treatment, don’t have dementia and AIDS.	tandem mass spectrometry for visit 1, radioimmunoassay for visit 2	25OHD serum concentration	3.0 Tesla scanner, (Siemens Tim-Trio, Erlangen, Germany), Analyzed by VBM	Total brain volume(mm³), Grey matter volume(mm³), white matter volume (mm³),	8/11

In terms of methods and outcomes, serum 25(OH)D (Brouwer-Brolsma et al., 2015, Beydoun et al., 2020) or S-25(OH)D (Berg et al., 2018) concentrations were measured using radioimmunoassay (Berg et al., 2018, Beydoun et al., 2020) and LC-MS/MS (Brouwer-Brolsma et al., 2015, Beydoun et al., 2020). Beydoun et al. also assessed folate and cobalamin levels (Beydoun et al., 2020)

Brain morphometry, including total brain, gray matter, and white matter volumes, was assessed by 3-Tesla MRI scanner (Brouwer-Brolsma et al., 2015, Berg et al., 2018, Beydoun et al., 2020). As indicated in Table 2, MRI data analysis was performed using FSL-VBM (Brouwer-Brolsma et al., 2015, Berg et al., 2018), FSPGR (Berg et al., 2018), and VBM (Brouwer-Brolsma et al., 2015, Beydoun et al., 2020) software.

The study populations consisted of 184 participants (44 % female) (Berg et al., 2018), 217 (43 % female) (Brouwer-Brolsma et al., 2015), and 240 (59 % female) (Beydoun et al., 2020) participants. The average age were 72 ± 6 (Brouwer-Brolsma et al., 2015), 30.53 ± 9.42 (Berg et al., 2018), and 47.7 ± 8.9 (Beydoun et al., 2020) years. Beydoun et al. focused on middle-aged White and African-American urban adults who were not pregnant and had no history of dementia, cancer treatment, or AIDS (Beydoun et al., 2020). Moreover, Brouwer-Brolsma et al. used data from an RCT study on preventing osteoporotic fractures in a geriatric population (≥ 65 years) with mild hyperhomocysteinemia (Brouwer-Brolsma et al., 2015). Berg et al. focused on younger and middle-aged adults without a history of drug use, organic etiology, or severe trauma to the central nervous system. Participants in study had an intelligence quotient above 70 and were proficient in Nordic languages (Berg et al., 2018).

Despite the studies included in the previous review (Annweiler et al., 2015), the studies in this meta-analysis considered genotypic variation. Berg et al. detected and analyzed genomic DNA, reporting an association between nine single-nucleotide polymorphism (SNP) markers of the vitamin D receptor (VDR) and vitamin D levels. They excluded participants not belonging to European ancestry to eliminate the impact of anthropometric variations as confounding factors (Berg et al., 2018).

### Results of quality assessment

Using the Newcastle–Ottawa quality assessment criteria, the included studies showed a low risk of bias, with scores of 8/8 in two studies (Brouwer-Brolsma et al., 2015, Berg et al., 2018) and 8/11 scores for the third (Beydoun et al., 2020).

### Vitamin D and whole brain volume

In the study by Brouwer-Brolsma et al., variables such as BMI, age, gender, education, smoking habits, alcohol consumption, season, depressive symptoms, and physical activity were identified as confounding factors. They showed a significant correlation between total brain volume and serum 25(OH)D (β ± SE: 0.26 ± 0.11 mL; P = 0.02) after intracranial volume adjustment. However, after adjusting for all confounding factors, no significant association was indicated (Brouwer-Brolsma et al., 2015).

According to Berg et al., serum 25(OH)D levels were associated with normalized whole brain volume after adjusting for confounding factors such as age, gender, and height (p < 0.02) (Berg et al., 2018).

In Beydoun et al.'s study, a direct correlation was detected between 25(OH)D and whole brain volume in the total sample, as well as in male and older subgroups (Beydoun et al., 2020).

### Vitamin D and gray matter volume

Brouwer-Brolsma et al. demonstrated a positive correlation between gray matter volume and serum 25(OH)D levels. After adjusting for confounding factors, gray matter volume increased by 0.20 mL (0.01 %) for every 1 nmol/L increase in serum 25(OH)D (Brouwer-Brolsma et al., 2015). In contrast, Berg et al.’s study found no correlation between vitamin D and peripheral gray matter volume (Berg et al., 2018).

### Vitamin D and white matter volume

Brouwer-Brolsma et al. observed no considerable change in white matter volume (Brouwer-Brolsma et al., 2015). Based on Berg et al.’s research, no significant association was found between serum vitamin D levels and white matter volume (Berg et al., 2018). Beydoun et al. concluded that vitamin D level is associated with higher white matter volumes in the occipital and parietal regions (overall: β = +910 ± 336, p = 0.007, q = 0.067) and passed the Bonferroni correction with an effect size of b= 0.19. Moreover, a greater effect size (b = 0.41) was observed in male participants (Beydoun et al., 2020).

### Meta-analysis

Due to noticeable differences in study designs, we used both random-effects and fixed-effects models. The findings indicated no significant association between vitamin D levels and brain volumetric changes.

**Fig. 2:**
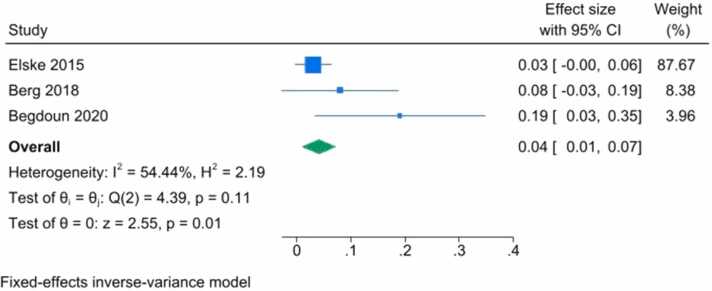
Forest plot of fixed effects inverse-variance model for correlation between serum vitamin D level with brain volumetric changes showed a small significant effect size of 0.04 (0.01, 0.07) with P-value of 0.01. however, there was a severe heterogenicity (I2 =54.44 %).

The fixed-effects model indicated small-study effects (overall effect size = 0.04, p = 0.01, 95 % CI = [0.01, 0.07]). Among the studies, Beydoun et al. and Brouwer-Brolsma et al. had the highest and lowest effect sizes, respectively (Fig. 2). However, due to severe heterogeneity (I^2^=%54.44), a random-effects model analysis was required.

**Fig. 3:**
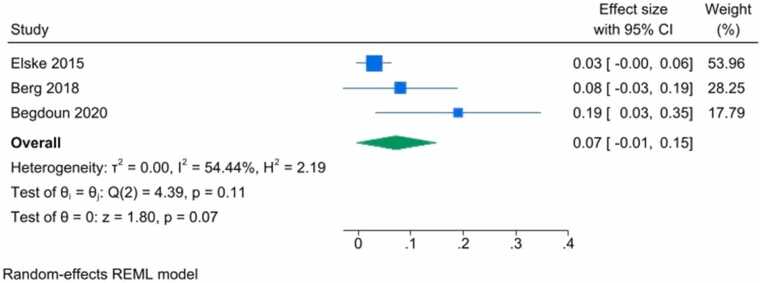
Forest plot of random effects REMIL model demonstrated a non-significant association between serum vitamin D level with brain volumetric changes with an effect size of 0.07 (-0.01, 0.15) (P-value=0.07).

The random-effects model demonstrated a non-significant result (overall effect size: 0.07, p = 0.07, 95 % CI = [-0.01, 0.15]), suggesting that vitamin D level does not have a considerable effect on brain volumetric changes (Fig. 3).

**Fig. 4:**
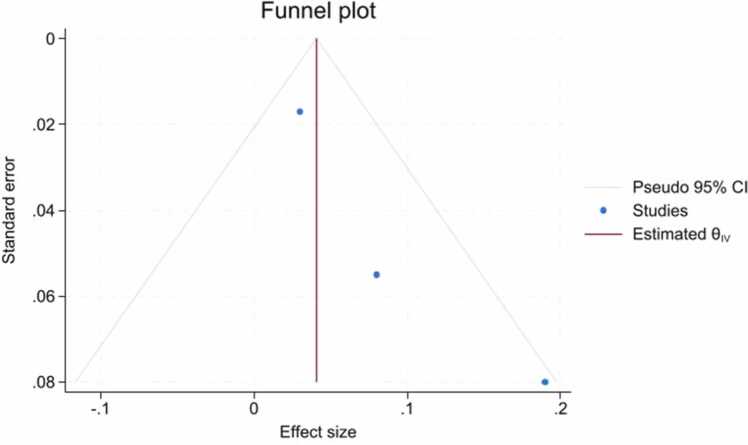
The funnel plot showed a symmetric pattern.

The result of Egger's test was statistically significant, suggesting the presence of publication bias in the studies. Additionally, the result of Begg's test was not statistically significant. However, the funnel plot showed a symmetric pattern (Fig. 4).

## Discussion

As the p-values are below 0.1, the results indicate a trend consistent with previous studies. Therefore, it is worth noting the consistent tendency, even though the findings do not reach statistical significance, likely due to the sample size.

Based on the analysis of three included studies with a total of 241 participants, our findings suggest no significant association between vitamin D levels and brain volumetric changes. Initially, it could be suggested that brain atrophy and the associated loss of autonomy result in inadequate dietary intake of vitamin D and reduced sun exposure, leading to a subsequent vitamin D deficiency (Annweiler et al., 2011, Annweiler and Beauchet, 2011). However, this hypothesis is not yet supported, as clinical studies have found no significant differences in body mass index or autonomy between participants with and without hypovitaminosis D (Annweiler et al., 2013). Moreover, in preclinical studies, vitamin D depletion was experimentally induced before the onset of brain changes (Eyles et al., 2003, Feron et al., 2005).

Beydoun et al. utilized two prospective waves from the Healthy Aging in Neighborhoods of Diversity Across the Life Span (HANDLS) study, conducted in Baltimore, Maryland (2004–2015), which included 183–240 urban adults aged 30–64 years in the first wave. This study is one of the few to use a brain scan-wide analysis approach to examine the relationships between serum 25(OH)D, folate, and cobalamin levels with brain volumes and white matter integrity (WMI). It is the first to investigate these associations in a socio-demographically diverse adult population. The three vitamin status indicators were systematically linked to structural MRI and diffusion MRI brain markers across varying segmentation levels. Our findings revealed statistically significant (FWER < 0.05) positive correlations between 25(OH)D(v1) and total, occipital, and parietal white matter volumes, particularly among men and older individuals, as well as with the left occipital pole volume, overall and more so in participants living above the poverty line (Beydoun et al., 2020).

Berg et al. investigated the relationship between vitamin D levels and brain phenotypes in psychotic disorders. Their study also examined potential interactions with genetic variants, such as those in the vitamin D receptor (VDR) and other genes involved in regulating vitamin D levels in the body. The researchers analyzed a cohort comprising 83 individuals diagnosed with psychosis and 101 healthy controls. Vitamin D levels were evaluated through measurements of serum 25-hydroxyvitamin D, while genotyping and structural magnetic resonance imaging were employed for neuroimaging analysis. They did not find an association between serum 25-hydroxyvitamin D [S-25(OH)D] levels and total ventricular volume. Whole brain and white matter volumes showed a significant association with the interaction between the VDR *Bsm*I marker rs1544410 and patient status, but not with current serum 25-hydroxyvitamin D [S-25(OH)D] levels. Ventricular size was predicted by the CYP24A1 SNP rs6013897 but was not associated with current serum 25-hydroxyvitamin D [S-25(OH)D] levels. Peripheral grey matter volume was the only brain phenotype in this study linked to serum 25-hydroxyvitamin D [S-25(OH)D] levels, and this association was observed only in patients (Berg et al., 2018).

In a study conducted by Brouwer-Brolsma et al., serum 25(OH)D, plasma glucose, and insulin were assessed as exposure variables, while total brain volume, gray matter volume, and white matter volume were measured using MRI as outcome variables. The study examined the relationships of serum 25(OH)D, plasma glucose, and insulin concentrations with brain tissue volumes through multiple linear regression analyses. Additionally, the potential moderating effect of glucose homeostasis on the association between 25(OH)D and brain volumetric measures was explored through stratification and testing for interaction. Higher serum 25(OH)D levels and lower plasma glucose are correlated with larger gray matter volume, but not with white matter or total brain volume, in a population of adults aged 65 and older in the Netherlands (Brouwer-Brolsma et al., 2015).

In a systematic review and meta-analysis conducted by Annweiler et al., evidence demonstrated that vitamin D deficiency is associated with reduced brain volume, particularly with enlarged lateral ventricles. The results indicated a strong association between vitamin D depletion and the expansion of the cerebral lateral ventricles, with ventricle volume being 1.0 standard deviation (SD) higher in participants with vitamin D deficiency compared to others (Fig. 2). Results for brain subvolumes were more mixed, suggesting that the brain atrophy observed in the case of vitamin D depletion might be attributed not to temporal lobe atrophy but rather to a loss of matter at the cranial vertex, possibly in the precuneus cortex (Annweiler et al., 2014).

This finding aligns with in vivo studies showing that rats born to vitamin D-deficient mothers had larger lateral ventricles compared to controls. This enlargement was attributed to brain tissue atrophy, likely caused by reduced mitotic cell proliferation due to decreased levels of NGF and GDNF in the brains of vitamin D-depleted newborns (Eyles et al., 2003, Feron et al., 2005).

One of the main limitations of our study is the relatively small number of studies included in the analysis, which may have contributed to insufficient statistical power. As a result, we were unable to establish a significant association between vitamin D levels and volumetric changes. The limited sample size may have restricted our ability to detect meaningful correlations, and future research with a larger number of studies and participants is needed to better assess these potential relationships. Additionally, the scope of existing studies is geographically restricted, as all included research was conducted in high-income countries (USA, Norway, Netherlands). This limitation further reduces the generalizability of our findings, as socioeconomic, genetic, and environmental factors unique to these regions may influence vitamin D metabolism and brain health. Expanding research to include diverse populations across different geographic and economic backgrounds will be crucial for validating these associations and ensuring broader applicability of the results.

## Conclusion

**Fig. 5:**
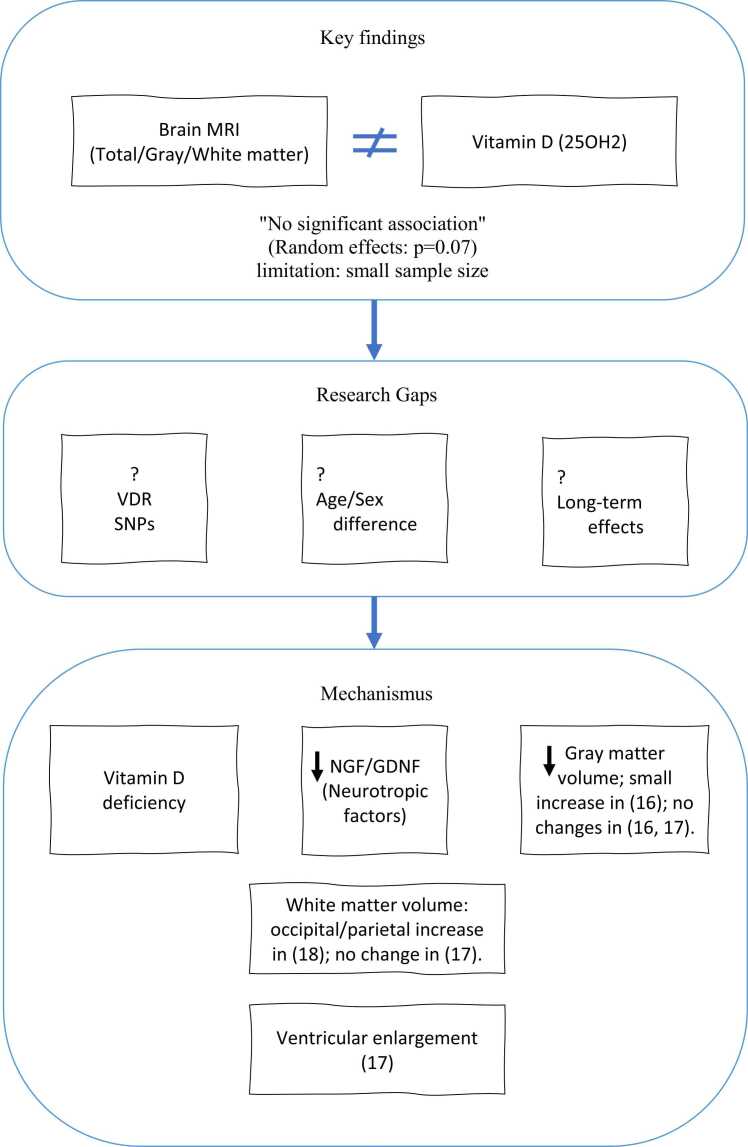
Summary of findings and future directions on vitamin D and brain volumetric; ≠ :not associated, ?:not clarified, ↓: decrease.

While our meta-analysis found no significant association between vitamin D and brain volumetric changes, the limited statistical power (due to small sample size) and between-study heterogeneity (I² = 54.44 %) preclude definitive conclusions. To better illustrate the findings and research gaps in this field, a schematic figure (Fig. 5) has been provided in this paper. This figure visually demonstrates that vitamin D levels are not significantly associated with brain volumetric changes and highlights areas where further research is warranted. Further larger, homogeneous prospective studies are needed to better assess and validate this potential relationship of vitamin D and brain volumetric changes.

## CRediT authorship contribution statement

**Aghazadeh-Habashi Komeil:** Resources. **Nozad Varjovi Mahdiyeh:** Writing – review & editing, Software, Methodology, Conceptualization. **Sarlak Hadis:** Writing – original draft. **Bayani Maedeh:** Writing – review & editing, Writing – original draft, Conceptualization. **Ahanj Faezeh:** Conceptualization. **Rahmani Sana:** Writing – review & editing, Writing – original draft, Conceptualization. **Hajihosseini Sajjad:** Methodology. **Yousefzadeh Salar:** Writing – review & editing, Writing – original draft, Conceptualization. **Deravi Niloofar:** Writing – review & editing, Writing – original draft, Visualization, Validation, Supervision, Software, Resources, Project administration, Methodology, Investigation, Funding acquisition, Formal analysis, Data curation, Conceptualization. **tamannaeifar rozhina:** Writing – review & editing, Writing – original draft, Supervision, Resources, Methodology, Conceptualization. **Khakpour Yaser:** Writing – review & editing, Writing – original draft. **Alizadeh Alaleh:** Writing – review & editing, Writing – original draft. **Arab Bafrani Melika:** Writing – review & editing, Writing – original draft. **Kohansal Vajargah Mahsa:** Writing – review & editing, Writing – original draft. **Ranjkesh Mona:** Writing – review & editing, Writing – original draft. **Eftekhari Nima:** Writing – review & editing, Writing – original draft, Conceptualization.

## Conflicts of Interest

The authors declare that they have no known competing financial interests or personal relationships that could have appeared to influence the work reported in this paper.
